# The paradigm of somatic mosaicism in complex diseases

**DOI:** 10.18699/vjgb-26-68

**Published:** 2026-07

**Authors:** A.A. Sleptcov, M.S. Nazarenko, V.P. Puzyrev

**Affiliations:** Research Institute of Medical Genetics, Tomsk National Research Medical Center of the Russian Academy of Sciences, Tomsk, Russia Tyumen Cardiology Research Center – Branch of Tomsk National Research Medical Center of the Russian Academy of Sciences, Tyumen, Russia; Research Institute of Medical Genetics, Tomsk National Research Medical Center of the Russian Academy of Sciences, Tomsk, Russia Tyumen Cardiology Research Center – Branch of Tomsk National Research Medical Center of the Russian Academy of Sciences, Tyumen, Russia; Research Institute of Medical Genetics, Tomsk National Research Medical Center of the Russian Academy of Sciences, Tomsk, Russia

**Keywords:** somatic mutations, cardiovascular diseases, clonal hematopoiesis, сomplex/common diseases, соматические мутации, сердечно-сосудистые заболевания, клональный гемопоэз, многофакторные заболевания

## Abstract

The multifactorial etiology of complex diseases involves the interplay of polygenic/oligogenic susceptibility loci and environmental factors. Complex diseases are characterized by pronounced phenotypic variability, genetic heterogeneity, pleiotropy of genetic variants, variable penetrance, and expressivity. Advances in population-wide genomic sequencing have expanded our understanding of the genetic architecture of complex diseases significantly; however, germline variants explain only a fraction of the observed phenotypic variability. The introduction of deep DNA sequencing and single-cell multi-omics analysis has revealed an additional, previously underestimated category of risk factors: somatic mutations that continuously accumulate in cells throughout an individual’s lifespan, giving rise to genetic mosaicism. This review considers the role of somatic mutations in the pathogenesis of complex diseases in the context of their combined contribution with germline determinants to the formation of disease phenotypes. Particular attention is paid to clonal hematopoiesis of indeterminate potential as the best-studied model of somatic mosaicism associated with age-related conditions. It is demonstrated that the interaction of somatic and germline variants occurs within specific tissue contexts through mechanisms of clonal selection, stochastic clonal drift, and epigenetic dysregulation, causing organ dysfunction. Within the concept of somatic mosaicism, this work suggests a selection mechanism alternative to classical oncogenesis. This pathway, defined as “passive clonal dominance”, describes selection through the persistence of clones that gain advantage not via proliferation but through resistance to apoptosis under chronic stress, which may be particularly relevant to post-mitotic tissues such as the heart muscle and nervous tissue. From a practical standpoint, the somatic variants discussed herein are of interest as candidate biomarkers for predictive diagnostics and risk stratification of complex diseases, pending clinical validation. Moreover, they point to pathophysiological pathways that may reveal targets for therapies aimed at modulating clonal composition and preventing disease progression. The paper also discusses prospects for studying somatic mosaicism in the context of complex diseases, including the potential of dynamic lineage tracing technologies for experimental verification of the proposed clonal selection mechanisms, as well as the need to integrate somatic and germline genetic variant data into unified models for individual disease risk assessment.

## Abstract (ru)

Многофакторные заболевания (МФЗ) характеризуются сложной этиологией, включающей взаимодействие полигенных/олигогенных наследственных детерминант и факторов среды, а также выраженным клиническим полиморфизмом, генетической гетерогенностью, плейотропией генетических вариантов, вариабельной пенетрантностью и экспрессивностью. Достижения в области секвенирования геномов популяций существенно расширили понимание наследственной архитектуры МФЗ, однако герминативные варианты объясняют лишь часть наблюдаемой фенотипической вариабельности. Внедрение методов глубокого секвенирования ДНК и мультиомиксного анализа отдельных клеток позволило выявить дополнительную, ранее недооцененную категорию факторов риска, а именно соматические мутации, непрерывно накапливающиеся в клетках на протяжении онтогенеза и формирующие мозаичную генетическую конституцию организма (соматический мозаицизм). В настоящем обзоре рассматривается роль соматических мутаций в этиопатогенезе МФЗ с позиции их сочетанного вклада с герминативными детерминантами в формирование патологического фенотипа. Особое внимание уделено феномену клонального гемопоэза неопределенного потенциала как наиболее изученной модели соматического мозаицизма, ассоциированного с возраст-зависимой патологией. Показано, что взаимодействие соматических и герминативных вариантов реализуется в клетках в специфических тканевых контекстах через механизмы клональной селекции, стохастического клонального дрейфа и эпигенетической дисрегуляции, приводящих к дисфункции органов. В рамках концепции соматического мозаицизма в работе обосновывается механизм селекции, альтернативный классическому онкогенезу. Данный путь определяется как «пассивная клональная доминантность» и описывает селекцию как персистенцию клонов, получивших преимущество не за счет пролиферации, а благодаря резистентности к апоптозу в условиях хронического стресса. Такой механизм может быть особенно важен для постмитотических тканей, таких как миокард и нервная ткань. С практической точки зрения рассмотренные соматические варианты представляют потенциальный интерес в качестве кандидатных биомаркеров для предиктивной диагностики и стратификации риска МФЗ, что требует дальнейшей клинической валидации. Более того, они указывают на патофизиологические пути, потенциально открывающие мишени для таргетной терапии, направленной на модуляцию клонального состава и предотвращение прогрессирования заболеваний. В заключение даны перспективы и направления изучения соматического мозаицизма в контексте МФЗ, включая потенциал технологий динамического отслеживания клеточных линий для экспериментальной верификации предложенных механизмов клональной селекции. Отмечена важность интеграции данных о соматических и герминативных генетических вариантах в единые модели оценки индивидуального риска заболеваний.

## Introduction

Complex diseases (CDs) are characterized by high prevalence and broad phenotypic diversity across populations. Although these disorders do not follow classic Mendelian inheritance patterns, familial clustering is well documented and is best explained by polygenic or oligogenic models incorporating substantial environmental contributions. Importantly, the balance between genetic susceptibility and environmental exposures varies across individuals, even within the same diagnostic category.

The phenotypic manifestation of CDs reflects core genetic principles, including clinical heterogeneity, locus heterogeneity, extensive pleiotropy of risk variants, and variable penetrance and expressivity, each shaped by the broad polygenic architecture and gene-environment interactions.

Beyond this genetic complexity, the disease phenotype in complex disorders exhibits multilayered heterogeneity spanning molecular, cellular, and tissue levels. Disease trajectories are dynamic: they evolve over time, and display spatial specificity driven by variation in local tissue microenvironments and cell-type-specific transcriptional programs. Substantial variability is observed among patients diagnosed with the same CDs, both in clinical presentation and in underlying pathogenic mechanisms.

Unlike inherited germline variants, somatic mutations arise de novo and accumulate continuously throughout the lifespan. The dynamics of this accumulation ranges from approximately linear in postmitotic tissues to markedly nonlinear in the setting of clonal selection (Lodato et al., 2018; Brunner et al., 2019). This ongoing mutagenesis generates intraorganismal genetic diversity, or somatic mosaicism, which is a form of postzygotic variation that can shape individual phenotypic outcomes. The accompanying epigenetic drift across clonal populations may further amplify phenotypic heterogeneity.This review addresses the role of somatic mosaicism in CD pathogenesis. We focus on somatic variants as tissue-specific modifiers of hereditary risk and as autonomous drivers of disease initiation and progression via clonal selection, stochastic clonal drift, and epigenetic dysregulation, which remodel the clonal architecture of affected tissues.

## Somatic genetic variants and complex diseases

During embryogenesis, the high rate of cell divisions, coupled with shortened cell cycles and relaxed checkpoint control, leads to the accumulation of de novo mutations. When a progenitor cell carrying such a mutation remains viable and maintains its proliferative potential, it gives rise to a clone whose genome diverges from the constitutional germline genome

In adult organisms, somatic mutations arise ubiquitously, but their accumulation and clonal expansion occur predominantly in tissues with high cellular turnover, such as the hematopoietic system and barrier epithelia, or in parenchymal organs under conditions of chronic injury and regeneration. Recurrent cycles of cell division increase the likelihood of acquiring somatic mutations, among which driver mutations in genes that directly regulate the cell cycle, proliferation, and cell survival are of particular importance.

Concurrently, neutral passenger mutations accumulate without directly affecting cell fitness. However, some mutations that do not meet the classic definition of oncogenic drivers may still exert indirect effects on cell survival through modulation of metabolic pathways, stress responses, or intercellular interactions within the microenvironment, thereby conferring a selective advantage in specific tissue environments.

Age-related decline in DNA repair efficiency contributes to the accumulation of somatic genetic variants, a subset of which confers a selective advantage to cells. As the regenerative capacity of tissues becomes depleted, this process drives clonal expansion of the most viable cell populations. Consequently, mosaic clonal populations emerge within tissues and bring the interindividual genetic diversity to such a degree that in some tissues it may exceed that observed at the population level (Harris, 2025).

Somatic mutagenesis has historically been a focus of cancer genomics research, as the accumulation of driver mutations in oncogenes and tumor-suppressor genes is considered pivotal in the pathogenesis of malignancy.

Advances in genomic technologies, including nextgeneration sequencing and single-cell genomics, have enabled the identification of nonmalignant complex diseases in which somatic mutations of various classes accumulate (see the Table).

**Table 1.**
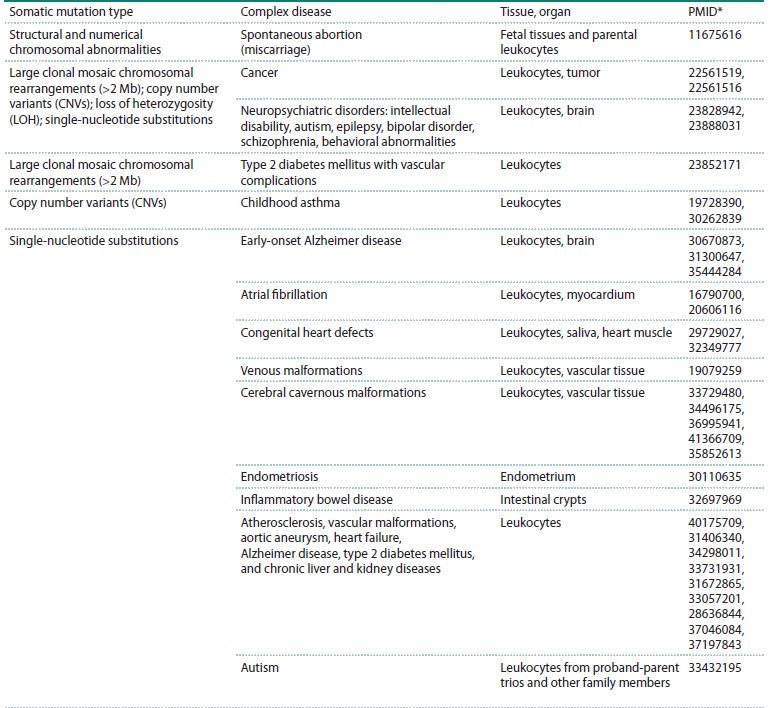
Somatic genetic variants in complex diseases * Pubmed ID.

A substantial fraction of postzygotic mutations likely remains undetected due to negative selection. Deleterious loss-of-function (LoF) variants in essential genes trigger apoptosis or irreversible cell cycle arrest in affected cells, rendering them invisible to standard genomic analysis methods.

Trio-based DNA sequencing (mother-father-child) enables the identification of de novo germline mutations. In contrast, detection of somatic variants requires comparative analysis of different tissues from the same individual, as well as the use of deep sequencing or single-cell sequencing technologies to detect low-level mosaicism (Shao et al., 2025).

Recent studies using single-cell genome sequencing have revised traditional views on the burden of somatic mutations in postmitotic tissues. Neurons from healthy adults have been shown to carry more than 1,000 somatic single-nucleotide variants (SNVs) per cell, markedly exceeding earlier estimates (Lodato et al., 2018).

Similarly, an analysis of 48 cardiac muscle cells from 10 healthy subjects of different ages has demonstrated that a single cardiac cell contains 4,000 to 30,000 somatic SNVs, with mutation burden showing a strong correlation with subject age (Choudhury et al., 2022). These findings, obtained through direct mutation counting in individual cells, indicate that somatic SNV accumulation is a widespread and substantial process in nonproliferating cells, potentially contributing to physiological aging and disease susceptibility.

Postzygotic mutations represent a fundamentally distinct, non-Mendelian source of phenotypic variability. They can not only modify the penetrance and expressivity of inherited predisposition but also initiate pathological processes independently through clonal selection mechanisms within target tissues. Thus, somatic mosaicism may function not only as a modifier of inherited risk but also as an independent pathogenic factor contributing to disease development in specific clinical contexts.

Below, we discuss the role of somatic mutations in CDs using cardiovascular disorders as an example.

## Somatic genomic variability in cardiovascular diseases 

Somatic mutagenesis, which gives rise to tissue mosaicism, affects not only parenchymal cells of target organs but also blood cells. In the hematopoietic system, this process manifests itself as clonal hematopoiesis (CH), in which a hematopoietic stem cell (HSC) harboring a driver mutation acquires a selective advantage, initiating clonal expansion.

Of particular importance is the phenomenon of clonal hematopoiesis of indeterminate potential (CHIP), in which a clonal population of cells carrying somatic mutations in genes associated with hematologic malignancies is present in peripheral blood, in the absence of evidence for myelodysplasia, leukemia, or other hematologic neoplasm in the individual (Jaiswal et al., 2014; Khoury et al., 2022).

Extensive research of CHIP in recent years has not only established its status as an independent risk factor for CDs but also added much to our understanding of the pathogenic role of somatic mosaicism by demonstrating that mutant hematopoietic clones can induce a state of chronic sterile systemic inflammation.

The pathogenic significance of this phenomenon lies in the acquisition of aberrant proinflammatory activity by mutant immune cell clones (primarily macrophages). Their ability to overproduce cytokines establishes a state of low-grade chronic systemic inflammation. This process is considered the key mechanism through which CHIP contributes to increased risk of atherosclerosis, coronary artery disease, heart failure, and other agerelated disorders (Jaiswal et al., 2017).

It is important to note that the pathogenic impact of somatic mosaicism on the cardiovascular system is not confined to the hematopoietic compartment. An equally significant parallel mechanism is oligoclonal expansion of resident cells of the vascular wall, particularly smooth muscle cells. In response to injury or atherogenic stimuli, individual progenitor cells acquire a competitive advantage, forming clonal clusters within atherosclerotic plaques. This process fundamentally affects plaque stability and progression (Lin et al., 2023).

## Clonal hematopoiesis of indeterminate potential in cardiovascular disease: molecular mechanisms and key determinants

According to the consensus criteria proposed by D.P. Steensma et al. (2015), mutations in CHIP are defined by a variant allele frequency (VAF) of 2 % or greater. They occur in a relatively restricted set of hematopoietic driver genes, including DNMT3A, TET2, ASXL1, JAK2, and others (Steensma et al., 2015). We should note that this threshold reflects the sensitivity limit of standard next-generation sequencing and does not preclude the pathogenic relevance of clones with lower VAFs.

The prevalence of CHIP correlates with age. Using the standard detection threshold (VAF ≥ 2 %), prevalence reaches 15–20 % among individuals older than 70 years, whereas deep sequencing approaches with higher sensitivity reveal substantially higher frequencies of low-level clones. Although CHIP is not associated with clinically overt cytopenias, it shows associations with subclinical alterations in erythrocyte indices, most notably wider red blood cell distribution and larger mean corpuscular volume (Acuna-Hidalgo et al., 2017; Walsh et al., 2022).

In individuals with CHIP, the relative risk of developing hematologic malignancies increases approximately 10- to 13-fold across multiple cohort studies, although this estimate varies considerably depending on clone size and the spectrum of driver mutations (Genovese et al., 2014; Jaiswal et al., 2014).

The risk of clinically overt coronary artery disease (CAD) depends on a specific mutated driver gene in CHIP. Mutations in DNMT3A, TET2, or ASXL1 are associated with a double risk of CAD, whereas the carriership of the JAK2 p.Val617Phe mutation is associated with a 12-fold increase in relative risk, an effect presumably mediated by a concomitant subclinical myeloproliferative phenotype (Jaiswal et al., 2017).

A direct correlation has been established between mutant clone size (VAF) and the atherosclerotic burden, as assessed by the coronary artery calcium score (Agatston score). Among patients with CHIP, a high frequency of ischemic stroke and other cardiovascular events is observed, which increases all-cause mortality in this group.

Although the panel of genes associated with CHIP includes up to approximately 100 loci, mutations in DNMT3A, TET2, and ASXL1 account for the majority of cases. They are identified in a substantial proportion of CAD patients with CHIP (Jaiswal et al., 2017). Lossof- function mutations in DNMT3A and TET2 in mouse models lead to disruption of epigenetic control, resulting in enhanced HSC self-renewal, impaired differentia-tion, and subsequent clonal dominance in the hematopoietic system (Challen et al., 2011; Moran-Crusio et al., 2011).

The molecular pathogenesis of DNMT3A mutations involves focal hypomethylation of specific genomic loci and subsequent aberrant derepression of genes that regulate HSC self-renewal, including the Hox cluster (HOXA5, HOXA7, and HOXA9). Loss of TET2 function impairs oxidative demethylation, leading to focal persistence of 5-methylcytosine in regulatory regions of the genome, which disrupts the enhancer landscape and blocks terminal differentiation of myeloid progenitors (Challen et al., 2011; Moran-Crusio et al., 2011).

Although histone modification mediated by ASXL1 protein is recognized as a fundamental process required for physiological hematopoiesis, the detailed molecular mechanisms that confer a selective advantage to mutant cells in the setting of age-related CH remain to be insufficiently elucidated (Yamamoto et al., 2021)

Whereas wild-type ASXL1 maintains precise epigenetic control through regulation of repressive complexes, the pathogenesis of CHIP is closely linked to specific mutations, predominantly frameshifts in exon 12. Mutations in ASXL1 are known to produce a stable truncated protein (ASXL1-trunc) with gain-of-function properties. This protein intensely destabilizes the PRC2 complex while simultaneously enhancing the interaction with BAP1 deubiquitinase within the PR-DUB complex. The synergistic effect of these activities leads to global reduction in H3K27me3 levels and aberrant derepression of critical leukemogenic loci, including HOXA cluster genes, which may confer a selective advantage to mutant cells (Inoue et al., 2016; Nagase et al., 2018; Yang et al., 2018).

Somatic mutations in JAK2, PPM1D, and TP53 associated with CHIP contribute to pathogenesis when occur in patients with cardiovascular diseases. In contrast to TET2 and DNMT3A, mutations in TP53 and PPM1D often emerge under selective pressure, such as chemotherapy, and they are associated with a more aggressive clone. The JAK2 tyrosine kinase acts as a key component of the JAK–STAT signaling pathway. The presence of somatic mutations induces constitutive kinase domain activity, resulting in cytokine-independent proliferation and conferring a selective advantage to the mutant clone (Kralovics et al., 2005).

The TP53-encoded p53 protein serves as a central regulator of the response to DNA damage (DDR) and cellular stress. Hematopoietic stem cells carrying loss-offunction mutations in TP53 acquire a selective advantage by evading apoptosis and escaping cellular senescence, leading to clonal dominance under conditions of competitive interaction with wild-type cells (Bondar, Medzhitov, 2010). An additional factor contributing to the clonal advantage of TP53-mutant cells may be higher tolerance to oxidative stress and genotoxic impacts. PPM1D also functions within the DDR pathway as a downregulator of p53, and truncating mutations in exon 6 provide a proliferative advantage to HSCs through enhanced resistance to p53-mediated apoptosis (Hsu et al., 2018).

Although CHIP mutations are detected in somatic cells, mounting evidence demonstrates the existence of inherited predisposition to their acquisition in HSCs. In 2009, a genome-wide association study (GWAS) identified an association between a single-nucleotide variant (rs10974944) marking the JAK2 46/1 haplotype and predisposition to acquiring the somatic JAK2 p.Val617Phe mutation, as well as subsequent development of myeloproliferative neoplasms (Kilpivaara et al., 2009).

It was found later that the risk of CHIP is tightly associated with genetic variants in the TERT gene (in particular, rs144418061), which encodes the catalytic component of the telomerase complex required for telomere maintenance, and with a polymorphism at the TCL1A locus (rs2887399) (Bick et al., 2020).

Furthermore, genetic analysis has revealed partial overlap between loci that predispose to acquisition of CHIP mutations (termed heritability of somatic mutation predisposition) and loci associated with cardiovascular disease risk in GWAS. This observation supports the existence of shared germline determinants that modulate individual risk profiles by simultaneously affecting the kinetics of mutant HSC clonal expansion and vascular resilience through independent pathogenic pathways, exemplifying genetic pleiotropy.

The kinetics of clonal expansion of mutant HSCs is tightly controlled by epigenetic regulatory mechanisms. A strong correlation between the growth rate of mutant clones and measures of epigenetic aging, assessed both through direct DNA methylation profiling and by using genetic predictors, has been confirmed. Markers of biological age, including the PhenoAge, GrimAge, and HannumAge algorithms, show positive associations with both the presence of CHIP and the rate of clonal hematopoietic cell proliferation (Nachun et al., 2021; Mack et al., 2024).

In addition to constitutional and endogenous determinants, exogenous stressors and proinflammatory settings significantly influence the dynamics of mutant HSC clonal expansion. Cigarette smoking and iatrogenic interventions, particularly cytotoxic therapy, increase the frequency of somatic mutations. Chronic systemic inflammation acts primarily as a selective pressure driving clonal outgrowth. Collectively, these processes create an adaptive advantage for clones that are resistant to apoptosis and cytotoxic effects.

The phenomenon of CH shows significant associations not only with progression of atherosclerosis and thromboembolic complications but also with a broad range of heterogeneous age-related conditions, positioning it as a key driver of the chronic systemic inflammation that accompanies aging (inflammaging). The range of comorbid disorders includes atrial fibrillation, heart failure, abdominal aortic aneurysm, venous thromboembolism, as well as Alzheimer’s disease-related neurodegeneration and metabolic disorders, particularly type 2 diabetes mellitus (Schuermans, Honigberg, 2025).

Studies in mouse models, first of all, bone marrow transplantation from Tet2 or Dnmt3a knockout mice, demonstrate that CHIP-associated clones of macrophages and other myeloid derivatives produce excessive amounts of proinflammatory cytokines and chemokines, such as IL-1β, IL-6, and CXCL2. This persistent proinflammatory signaling induces dysfunction in peripheral tissues and target organs, initiating the development of insulin resistance and disruption of tissue homeostasis. These pathophysiological shifts recapitulate the multisystem phenotype of accelerated aging observed in clinical practice (Schuermans, Honigberg, 2025).

A specific form of clonal mosaicism is mosaic loss of the Y chromosome (mLOY) in peripheral blood leukocytes, which represents the most common somatic event in men. This phenomenon is associated with increased risk of cardiovascular disease, particularly the development of cardiac fibrosis and heart failure (Sano et al., 2022), as well as with severe progression of neurodegenerative disorders (Ljungström et al., 2022). In men with pronounced mLOY, a trend toward increased VAF of CHIP-associated mutations is observed, suggesting that these events may coexist within the same clone. The available evidence indicates that CHIP and mLOY frequently co-occur during aging, supporting their consideration as potentially interconnected manifestations of common age-related changes in the clonal architecture of bone marrow (Ljungström et al., 2022).

Despite these associations, detailed elucidation of the molecular cascades linking CHIP to the broad spectrum of CDs requires further investigation. To explain mechanisms of cell selection, R. Happle (1993) put forward the concept of paradominance, which presumes that a somatic mutation arising in the setting of germline heterozygosity at the same locus leads to biallelic inactivation and local manifestation of a pathological phenotype during early development. However, the applicability of this model to CHIP driver genes such as TET2 or DNMT3A remains a matter of ongoing debate, because in this context the clonally significant phenotype often arises from monoallelic inactivation.

Nevertheless, the theoretical potential of this model lies in its ability to provide a mechanistic explanation for the phenomena of incomplete penetrance and variable expressivity in CDs (Puzyrev et al., 2014). This is particularly relevant for driver genes in which constitutional homozygous inactivation often results in severe developmental defects or is embryonic lethal.

## Conclusion and prospects of studying somatic mosaicism in complex diseases

Detailed research of the contribution of somatic mosaicism to CD etiopathogenesis opens prospects for the development of fundamentally new strategies for molecular diagnosis and therapeutic intervention. Such approaches should target not only the affected organ but also the selective elimination or reprogramming of specific pathogenic clones that contribute to disease progression, therapy resistance, and clinical heterogeneity. A fundamental requirement of this therapeutic paradigm is the necessity of accounting for the temporal dynamics of clonal architecture. The composition and population of pathogenic clones exhibit temporal instability, being subject to modification under selective pressures, including the cytotoxic effects of therapeutic agents.

Mechanisms of clonal dynamics are primarily considered through the lens of neutral drift, defined as stochastic replacement in the absence of selective pressure, as described in intestinal epithelium (Klein, Simons, 2011), or positive selection, which confers a proliferative advantage to mutant clones in renewing tissues such as the hematopoietic system. However, somatic mosaicism in postmitotic tissues, particularly the accumulation of mutations in neurons and cardiac muscle cells, is often interpreted merely as a passive process of genetic noise accumulation accompanying aging (Lodato et al., 2018). However, these frameworks do not fully account for the potentially nonrandom nature of mosaic landscape formation in tissues with extremely low proliferative capacity

In this review, we propose a conceptual model of an alternative mechanism for clonal selection in postmitotic tissues, which we term passive clonal dominance, realized through selection for persistence. This term refers to the increase in relative fraction of clones that do not possess a direct proliferative advantage but instead exhibit enhanced resistance to elimination under conditions of chronic stress

In proliferating tissues such as the hematopoietic system or epithelium, Darwinian selection favors clones harboring mutations that enhance proliferation and survival, commonly referred to as driver mutations. However, a fundamentally different scenario may occur in functionally specialized tissues with low or absent regenerative capacity, including populations of cardiac muscle cells and neurons. Under these conditions, the advantage is conferred not to proliferating clones but to populations of resilient cell populations characte- rized by enhanced resistance to elimination under chronic stress, so that the mosaic tissue architecture is remodeled, as cells with standard apoptotic thresholds are eliminated.

Such cellular selection may be driven by mutations that, within the specific microenvironment of postmitotic tissues, do not confer a direct proliferative advantage but instead promote cell survival. These include inactivation of genes controlling apoptosis, the integrated stress response (ISR), or metabolic homeostasis. In this model, the increased representation of a mutant clone is achieved not through mitotic activity but through its prolonged persistence, while neighboring wild-type cells with lower stress resistance undergo accelerated apoptosis.

A logical consequence of this process is the gradual accumulation of functionally impaired but eliminationresistant cellular clones, representing a distinct form of somatic evolution shaped by selective pressure from the microenvironment. This hypothetical process, through which tissues become enriched in clones with preserved viability but compromised function, may contribute to age-related tissue dysfunction and clinical manifestation of CDs alongside other well-established mechanisms. Experimental validation will require longitudinal studies and approaches for tracking clonal dynamics in vivo.

A key tool for such validation is dynamic lineage tracing, an approach that has already transformed our understanding of clonal architecture in hematopoiesis and can be adapted for the study of postmitotic tissues. Based on site-specific recombination systems (Cre-lox) combined with polychromatic reporter constructs, such as the Confetti or Rainbow systems, and high-throughput single-cell sequencing technologies (scRNA-seq), this approach has enabled direct observation of clonal expansion kinetics within tissue architecture (McKenna, Gagnon, 2019).

Our initial understanding of CHIP was shaped by largescale population studies using next-generation sequencing. The study of CHIP by lineage- tracing technologies have facilitated a shift from static detection of mutant clones to analysis of their dynamic history (Haghverdi, Ludwig, 2023; Jindal et al., 2024). In particular, it has become possible to determine the hierarchical origin of a clone by identifying the specific hematopoietic stem or progenitor cell that initiated expansion with mutations in DNMT3A, TET2, or ASXL1, and to reconstruct the complete phylogenetic tree of the clone within the bone marrow niche.

In addition, these methods enable real-time observation of competitive cellular dynamics, such as tracking how a clone carrying a driver mutation, e. g., JAK2 p.Val617Phe, outcompetes wild-type cells during aging or in response to proinflammatory stimuli (Haghverdi, Ludwig, 2023; Jindal et al., 2024). Finally, it has become possible to link clonal identity to its functional status, transcriptional profile, and epigenome at the single-cell level, thereby identifying determinants that account for the quiescence of some clones and the pronounced proinflammatory potential of others (Xie, Zeidan, 2023; Jindal et al., 2024).

Application of such integrated approaches to the study of somatic mosaicism is promising for transforming our knowledge of the molecular events through which somatic mutations reprogram the function of hemato- poietic and other somatic cells and thereby induce systemic inflammation and increase the risk of CDs (Jindal et al., 2024). Elucidating the mechanisms of clonal selection, including the proposed model of passive clonal dominance, provides grounds for the development of targeted interventions aimed at modulating tissue clonal architecture, which in turn may contribute to the prevention of a broad range of age-related conditions.

## Conflict of interest

The authors declare no conflict of interest.
